# Geographic and Institutional Patterns of Transoral Robotic Surgery in Head and Neck Cancer

**DOI:** 10.1002/hed.70179

**Published:** 2026-01-20

**Authors:** Daniel Gilmore, Lauren R. Michelle, Xiaodan Hu, Stephen Y. Kang, Nolan B. Seim, Catherine T. Haring, Matthew O. Old, Amit Agrawal, Enver Ozer, Lauren E. Miller

**Affiliations:** ^1^ The Center for the Advancement of Team Science, Analytics, and Systems Thinking in Health Services and Implementation Science Research (CATALYST), College of Medicine, The Ohio State University Columbus Ohio USA; ^2^ Department of Otolaryngology—Head and Neck Surgery The Ohio State University Wexner Medical Center Columbus Ohio USA

## Abstract

**Introduction:**

Transoral robotic surgery (TORS) is increasingly used for oropharyngeal squamous cell carcinoma (OPSCC), yet national patterns of TORS availability for Medicare beneficiaries are not well defined. We characterized hospital type, geographic distribution, and market concentration of TORS.

**Methods:**

We conducted a retrospective cross‐sectional study of inpatient Medicare claims from 2017 to 2023, identifying OPSCC with ICD‐10‐CM codes and TORS with ICD‐10‐PCS codes including a robotic‐assistance qualifier. Claims were linked to inpatient prospective payment system files for hospital teaching status, disproportionate share hospital (DSH) percentage, urbanicity, and geographic labor market area (GLMA). We mapped county‐level procedure counts, calculated GLMA‐level Herfindahl–Hirschman Index (HHI), and used negative binomial regression to evaluate associations of hospital factors with TORS volume and inpatient length of stay (LOS).

**Results:**

We identified 2499 unique TORS procedures at 161 hospitals; 86.2% occurred at teaching hospitals, and annual volume rose 31% from 2017 to 2023. TORS use was geographically diffuse but locally concentrated: among 102 GLMAs with any TORS, 64.7% had HHI = 10 000 and 28.4% had HHI 5000–9999. Six GLMAs with > 100 procedures accounted for 33.6% of all cases and were predominantly teaching centers. Higher teaching intensity was associated with greater TORS use (incidence rate ratio [IRR]: 1.99, 95% CI: 1.63–2.45). LOS was longer in urban and rural hospitals versus metropolitan centers and shorter in high‐volume GLMAs (IRR: 0.82, 95% CI: 0.76–0.87).

**Conclusion:**

Among Medicare beneficiaries with OPSCC, TORS is concentrated in teaching hospitals and a few high‐volume markets, with shorter LOS in high‐volume regions, highlighting trade‐offs between centralization and access.

## 1 Introduction

Oropharyngeal squamous cell carcinoma (OPSCC) incidence has steadily risen over the past two decades, with substantial evolution in its surgical management [1]. The advent of transoral robotic surgery (TORS) and subsequent approval by the U.S. Food and Drug Administration accelerated national adoption of this minimally invasive surgical approach [2, 3, 4, 5]. Identifying surgical candidacy depends on multiple factors, but TORS is generally ideal for patients with early‐stage disease (T1–T2), favorable anatomy, and no strong pretreatment indications for both adjuvant radiation and chemotherapy [3]. Despite increasing use of TORS, national characterization of where TORS is performed remains limited.

Previous studies analyzing practice patterns have primarily been survey‐driven [6, 7] or have utilized the National Cancer Database with limited information on provider and hospital characteristics [2, 4, 8]. While these previous analyses have provided valuable contributions, they lack detail on the geographic distribution of care or the regional availability of TORS. Understanding where patients receive care is essential, as access to high‐volume centers and technologically equipped hospitals can influence surgical approach, perioperative outcomes, and equity in treatment delivery. To address these gaps, we used a national claims database to characterize hospital type, regional distribution, and annual case volume of TORS for Medicare beneficiaries with OPSCC.

## 2 Methods

### 2.1 Study Design and Data Sources

We performed a retrospective, cross‐sectional analysis of inpatient surgical encounters for OPSCC using 100% Medicare standard analytic files (SAF) from 2017 to 2023. The SAF are Medicare administrative claims datasets that provide encounter‐level inpatient claims, including diagnoses, procedures, billing hospital, and hospital county. We linked inpatient claims to hospital attributes via the Centers for Medicare and Medicaid Services (CMS) hospital identifier in the CMS Inpatient Prospective Payment System (IPPS) files for each fiscal year [9]. IPPS files provide hospital‐level information including teaching intensity (resident‐to‐bed ratio), disproportionate share hospital (DSH) proportion (as a proxy for extent of low‐income patients within that hospital), core‐based statistical area/urbanicity (metropolitan, urban, rural), and the geographic labor market area (GLMA), a CMS‐defined region used to adjust hospital payments for local wage levels and used here as a proxy for local hospital markets [10].

### 2.2 Cohort Definition

We identified adult inpatient stays with a principal diagnosis of OPSCC using ICD‐10‐CM codes consistent with neoplasms of the oropharynx. TORS procedures were flagged using a pre‐specified ICD‐10‐PCS algorithm combining extirpation or resection of oropharyngeal subsites with the robotic‐assistance qualifier (Supplement). Similarly, we characterized the tumor subsite (tonsil, base of tongue, unspecified pharynx) and extent of neck dissection (ipsilateral vs. bilateral) via ICD‐10‐PCS coding. Any hospital with only a single robotic procedure performed during a claim year was excluded from analysis within that year. Similarly, any case without the robotic qualifier was excluded. For Medicare beneficiaries with more than one inpatient claim in the study period, the claim with the earliest admission date was retained for analysis.

### 2.3 Outcomes

The primary descriptive outcomes were annual and cumulative TORS utilization among Medicare beneficiaries. Given our focus on market structure, we also examined how TORS procedures were distributed across hospitals and GLMAs. Our primary measures of market structure were GLMA‐level TORS volume and the Herfindahl–Hirschman Index (HHI), which summarizes the extent to which TORS volume is concentrated in one or a few hospitals within each labor market. Secondary descriptive outcomes included TORS anatomic subtype (i.e., primary site and neck dissection laterality), number of unique hospitals performing ≥ 2 TORS cases annually, state‐level utilization of TORS, the proportion of procedures completed at teaching hospitals (using binary receipt of indirect medical education funding as well as continuous resident‐to‐bed ratio) [11], urbanicity (metropolitan, urban, rural), and DSH percentage.

To depict where procedures were performed, we aggregated TORS counts by hospital county from the inpatient claim to produce a county‐level choropleth map showing the geographic distribution of sites of care. The HHI for each GLMA was based on hospital shares of TORS volume within that GLMA over the study period [12]. We calculated HHI as the sum of squared hospital market shares within each GLMA, where a hospital's share equals its percentage of TORS cases in that GLMA. We report HHI on the 0–10 000 scale (percent units): 10000 indicates that a single hospital performed all cases (monopoly), whereas lower values indicate greater competition (e.g., ≈5000 for two duopoly, with two hospitals each performing half of cases; ≈3333 for three equal‐share hospitals). Further modeled outcomes included TORS counts across the study period and inpatient length of stay (LOS).

### 2.4 Statistical Analysis

Continuous variables are reported as means (standard deviation) and categorical variables as counts (percentages). For multivariable analyses, we fit negative binomial regression models with a log link function to determine incidence rate ratios (IRR) for TORS counts and LOS. For hospital TORS use, the dependent variable was the yearly TORS count by hospital; independent variables of interest were resident‐to‐bed ratio (Model 1) and DSH percentage (Model 2), with claim year included as a covariate in each model. For LOS, the dependent variable was LOS in days, and the independent variable of interest was urbanicity (reference: metropolitan), adjusting for claim year (Model 3). We also modeled LOS where a binary variable for high vs. low GLMA volume was also included as a covariate (Model 4). County‐level frequencies of TORS were used to generate a chloropleth map of TORS utilization by county using publicly available Datawrapper and MapChart software [13]. Analyses were performed in SAS 9.4 (SAS Institute, Cary, NC).

## 3 Results

### 3.1 Cohort and Hospital‐Level Characteristics

A total of 2867 TORS cases were performed during the study period. After restricting to unique beneficiaries and hospitals with ≥ 2 TORS cases per hospital‐year, 2499 procedures remained for analysis. Across the study period, 161 unique hospitals and 430 unique surgeons performed TORS, spanning ≥ 84% of states each year. Most procedures (86.2%) occurred at teaching hospitals. Among teaching hospitals, mean resident to bed ratios ranged from 45% to 49% annually, corresponding to the middle third of all teaching hospitals, and mean DSH percentages ranged from 34% to 36%, also in the middle third by DSH of hospitals (Table 1). Annual TORS volume increased 31% over the study period, from 292 procedures in 2017 to 382 in 2023 (Table 1). The most common primary subsite was the palatine tonsil (39%) (Table 2).

**TABLE 1:** TORS procedures by hospital setting and region, 2017–2023.

	2017	2018	2019	2020	2021	2022	2023
TORS procedures (*N*%)^a^	292 (11.7)	366 (14.6)	376 (15.0)	365 (14.6)	361 (14.4)	357 (14.3)	382 (15.3)
Unique hospitals	70	79	84	82	74	71	75
Unique states	46	42	49	49	48	49	46
Completed at teaching hospital, *n* (%)^b^	258 (88.4)	321 (87.7)	339 (90.2)	325 (89.0)	311 (86.1)	293 (82.1)	308 (80.6)
Resident to bed ratio (M(SD))^c^, ^d^	0.45 (0.28)	0.45 (0.29)	0.46 (0.28)	0.45 (0.28)	0.46 (0.32)	0.49 (0.30)	0.48 (0.32)
Hospital disproportionate share (M(SD))	35.6 (12.9)	36.1 (14.2)	36.6 (11.7)	35.8 (12.6)	34.4 (13.1)	34.6 (12.3)	35.6 (12.2)
Geographic category^e^							
Metropolitan, *n* (%)^f^	182 (62.3)	236 (64.5)	195 (51.9)	205 (56.2)	331 (91.7)^h^	225 (63.0)	245 (64.1)
Urban^g^	86 (29.5)	101 (27.6)	154 (41.0)	133 (36.4)	0 (0)	95 (26.6)	84 (22.0)
Rural	3 (1.0)	4 (1.1)	2 (0.5)	8 (2.2)	4 (1.1)	4 (1.1%)	8 (2.1)
Unknown	21 (7.2)	25 (6.8)	25 (6.6)	19 (5.2)	26 (7.2)	33 (9.2)	45 (11.8)

^a^ Among hospitals that completed > 1 procedure within the year.

^b^ Indirect medical education > 0.

^c^ Mean (standard deviation).

^d^ Among unique teaching hospitals within each claim year.

^e^ Categories determined by provider's core based statistical area.

^f^ Metropolitan, > 1 000 000 population.

^g^ < 1 000 000 population.

^h^ 2021 data categorized as only urban versus rural without indication of large versus other urban. These data were placed into the metropolitan category in this table.

**TABLE 2:** TORS subsite by year.

	2017	2018	2019	2020	2021	2022	2023	Total
Primary site								
Tonsil	106	144	150	137	139	149	150	975 (39.0%)
Base of tongue	72	78	85	78	28	66	54	461 (18.4%)
Tonsil and base of tongue	44	45	43	50	43	44	65	334 (13.4%)
Pharynx NOS	70	99	98	100	97	98	113	675 (27.0%)
Necks								
Ipsilateral neck	254	306	320	314	306	312	338	2150 (86.0%)
Bilateral neck	38	60	56	51	55	45	44	349 (14.0%)
	292	366	376	365	361	357	382	2499

Abbreviation: NOS, not otherwise specified.

### 3.2 Geographic Distribution

**FIGURE 1:**
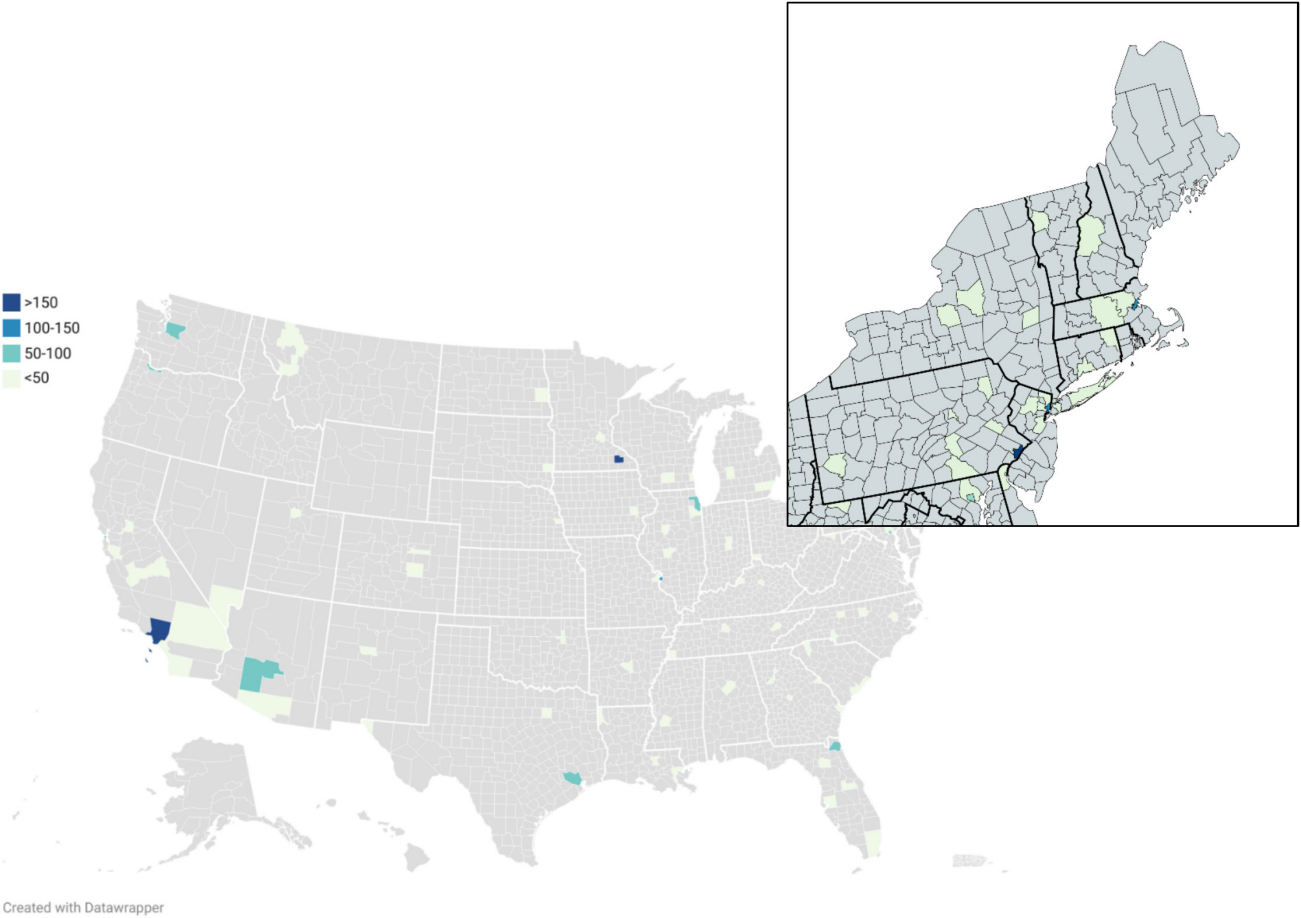
County‐level TORS utilization 2017–2023. [Color figure can be viewed at wileyonlinelibrary.com]

TORS use was geographically diffuse but highly concentrated within markets. At the county level, 87% of counties that performed TORS completed < 50 procedures during the study period (Figure 1). However, across 102 GLMAs represented in our data, 6 (5.9%) performed > 100 TORS procedures over the study period. These six markets comprised 26 unique hospitals, 25 of which (96.2%) were teaching hospitals, and each GLMA had 7–25 TORS surgeons. Collectively, these six GLMAs accounted for 33.6% of all TORS procedures for Medicare beneficiaries between 2017 and 2023 (Table 3).

**TABLE 3:** TORS procedures in highest volume GLMA regions, aggregate from 2017–2023.

GLMA *Corresponding urban region*	14 454 *Boston*,*MA*	41 180 *St. Louis, MO‐IL*	35 614 *New York‐Jersey City, NJ*	37 964 *Philadelphia, PA*	31 084 *Los Angeles, CA*	40 340 *Rochester, MN*
TORS procedures *N* (%)^a^	101 (4.0)	104 (4.2)	129 (5.2)	150 (6.0)	165 (6.6)	189 (7.6)
Surgeons (*N*)	15	11	25	14	16	7
TORS/surgeon	6.7	9.5	5.2	10.7	10.3	17.2
Hospitals (*N*)	5	3	9	3	5	1
Teaching hospitals (*N*%)	5 (100)	3 (100)	9 (100)	3 (100)	4 (80.0)	1 (100)
HHI^b^	4734	8217	3012	5208	4935	10 000

^a^ Percentage of total TORS procedures (2499).

^b^ Herfindahl–Hirschman index.

Across GLMAs with any TORS activity (*n* = 102), 66 (64.7%) exhibited HHI = 10 000, indicating a single hospital performed all TORS in that market, and 29 (28.4%) had HHI 5000–9999, consistent with duopoly‐level concentration. Only 7 (6.9%) had HHI < 5000. Overall, at least 92.7% of GLMAs were highly concentrated in one or two hospitals per labor market (exceeding the 2023 threshold of HHI > 1800). Among the six highest‐volume GLMAs, HHI values ranged from 3012 to 10 000.

### 3.3 Predictors of TORS Use

In negative binomial models adjusting for claim year, the rate of TORS use varied by hospital characteristics. Higher teaching intensity was associated with greater TORS use (incidence rate ratio [IRR]: 1.99; 95% CI: 1.63–2.45; *p* < 0.0001). In contrast, higher DSH percentage was associated with lower TORS use (IRR: 0.36; 95% CI: 0.22–0.60; *p* < 0.0001).

### 3.4 Length of Stay

Across all procedures, unadjusted mean LOS was 4.3 days (SD 5.9). In multivariable models controlling for claim year and hospital urbanicity, LOS was longer for procedures performed in urban CBSA areas (IRR: 1.13; 95% CI: 1.04–1.22; *p* = 0.002) and in rural areas (IRR: 1.64; 95% CI: 1.26–2.13; *p* < 0.001) compared with metropolitan hospitals. LOS was shorter for procedures performed in high volume GLMA (> 100 TORS procedures over the study period; IRR: 0.82; 95% CI: 0.76–0.87; *p* < 0.0001).

## 4 Discussion

This national analysis of Medicare claims provides the most comprehensive geographic characterization of TORS utilization for OPSCC to date and quantifies how TORS programs are structured across hospital labor markets. We found that, despite approximately 30% growth in procedure volume from 2017 to 2023, TORS remains predominantly concentrated in academic medical centers, with 86% of procedures performed at teaching hospitals and relatively few cases in rural settings.

Market structure analysis reinforced this concentration. In our cohort, 64.7% of GLMAs exhibited single‐hospital monopolies (HHI = 10 000) and an additional 28.4% showed duopoly‐level concentration. Taken together, more than 90% of TORS procedures stratified by GLMAs were in highly concentrated hospital markets. These patterns contrast with prior work in general surgery, where robotic surgery has been shown to diffuse rapidly across hospitals and procedures once programs are launched, rather than remaining anchored in a single hospital within a market [14]. The clustering of TORS within a small number of hospitals and markets likely reflects both structural and clinical factors. Survey data point to differences in practice patterns between fellowship‐trained and non–fellowship‐trained surgeons, regional variation, and barriers related to cost and credentialing [6, 15]. Given the capital investment, advanced training, and referral networks needed to sustain a TORS program, it is not surprising that use is concentrated in larger centers. We note that market concentration is conditional on the definition used; we used CMS GLMAs because they provide a stable, hospital‐linked geography available uniformly through IPPS linkage. Residence‐based hospital referral region assignment was not feasible because beneficiary residence ZIP/county was unavailable in our analytic extract.

These patterns have important implications. Concentration at high‐volume centers has been associated with improved outcomes in complex surgical procedures, including lower positive margin rates and reduced mortality in TORS [4]. Consistent with a volume–outcome relationship, the six highest‐volume GLMAs, accounting for one‐third of all Medicare TORS procedures, demonstrated characteristics consistent with centers of excellence: near‐universal teaching hospital status (96.2%), multiple TORS surgeons (7–25 per market), and sustained case volume. However, surgeon‐to‐procedure ratios within these markets varied dramatically (6.7–17.2 procedures per surgeon), suggesting heterogeneous practice patterns even within high‐volume environments. This raises questions about optimal case distribution and the minimum annual volume threshold needed to maintain competency [4, 15].

At the same time, centralization may exacerbate geographic disparities. Patients in rural areas must often travel long distances to reach high‐volume centers, and those unable to do so may receive alternative, potentially more morbid treatments or forego TORS entirely. In our cohort, TORS performed in rural hospitals was associated with a 64% longer adjusted length of stay (IRR: 1.64, 95% CI: 1.26–2.13) compared to metropolitan centers, which may reflect both differences in patient selection and less institutional experience. Balancing the benefits of centralization with equitable access is particularly important in OPSCC, where treatment is usually non‐emergent and allows time for referral, yet structural and socioeconomic barriers often persist. Because our cohort is limited to inpatient stays, LOS associations may be influenced by selection into inpatient admission, unmeasured clinical severity and complications, and local discharge practices.

This analysis leverages the comprehensiveness of Medicare claims to provide the first county‐level mapping of TORS utilization, capturing procedures across all hospital types rather than only Commission on Cancer‐accredited facilities. By linking to IPPS files, we could characterize hospital attributes and calculate market concentration metrics previously unavailable in TORS literature, allowing us to frame TORS as a regionally centralized service line rather than a hospital‐level technology. However, there are several limitations. First, our Medicare dataset captures primarily fee‐for‐service beneficiaries over 65, while HPV‐associated oropharyngeal cancer also affects younger patients [16, 17]. We excluded sites that performed only a single TORS procedure annually. As a result, our analysis underestimates the absolute number of TORS cases, and the geographic distribution of TORS may differ in commercially insured populations and in estimates that include all eligible beneficiaries. Cases where TORS was performed outpatient or observation status (including staged neck management) would not be captured, although requiring neck dissection may reduce under‐ascertainment for typical admitted surgical episodes. If outpatient/observation capture differs systematically by hospital type or geography, market concentration estimates could be biased; accordingly, HHI results should be interpreted within this inpatient, neck‐dissection–anchored framework. Second, claims data cannot assess clinical appropriateness—we cannot determine whether low TORS utilization in certain regions reflects lack of access or appropriate patient selection. Third, we cannot observe patients who were TORS candidates but received alternative treatments due to access barriers.

In summary, TORS for OPSCC remains concentrated in teaching hospitals within metropolitan areas, with most cases occurring in a few select regions. While this centralization may optimize outcomes for patients who access these centers, it creates substantial geographic disparities that may systematically disadvantage rural and underserved populations. Our findings provide essential data for health systems planning to expand access to minimally invasive surgical options while ensuring optimal oncologic outcomes.

## Funding

During this project Daniel Gilmore was supported by a National Institutes of Health (NIH) institutional training grant (1T32HS029590‐01). [Correction added after first online publication on 06 June 2026. Funding information has been updated.]

## Conflicts of Interest

The author declares no conflicts of interest.

## Supporting information


**Data S1:** Supporting information.

## Data Availability

The data that support the findings of this study are available on request from the corresponding author. The data are not publicly available due to privacy or ethical restrictions.
