# Supplementary material for: Geographic and Institutional Patterns of Transoral Robotic Surgery in Head and Neck Cancer
Source: Head Neck. 2026 Jan 20;48(6):1605–10. doi: 10.1002/hed.70179 (PMC13155175; doi:10.1002/hed.70179)
Supplement: Supplementary file 1 — Data S1: Supporting information. [file HED-48-1605-s001.docx]

**Supplement: ICD-10-PCS Coding Methodology**

To define the analytic cohort, encounters were required to have **at least one ICD-10-PCS code from each of the following three categories:** (1) robotic-assisted procedures, (2) primary tumor location, and (3) neck dissection.

1. Robotic-Assisted Procedures

- 8E097CZ, 8E090CZ8, 8E094CZ, 8E098CZ, 8E09XCZ

1. Primary Tumor Location

- 0CBM0ZZ, 0CBM7ZZ, 0CBM7ZX, 0CBM8ZX, 0CBM8ZZ, 0CBM4ZZ, 0CTM8ZZ, 0CTPXZZ, 0CTP0ZZ, 0CBPXZZ, 0CBPXZX, 0CB7XZZ, 0CB70ZZ, 0CT70ZZ

1. Neck Dissection

- 07T20ZZ, 07T10ZZ, 07B20ZZ, 07B10ZZ, 07B10ZX, 07B20ZX
